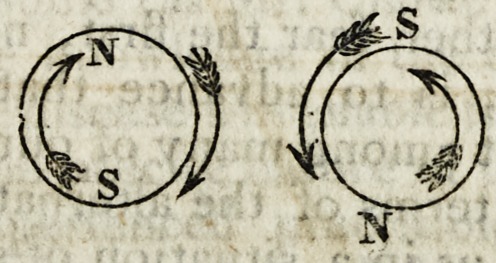# Collectanea Medica: Consisting of Anecdotes, Facts, Extracts, Illustrations, &c.

**Published:** 1822-01

**Authors:** 


### COLLECTANEA MEDICA:

CONSISTING OF

### ANECDOTES, FACTS, EXTRACTS, ILLUSTRATIONS, &c.

*Relating to the History or the Art of Medicine, and the Collateral Sciences.*

Floriferis, ut apes, in saltibus omnia libant,

Omnia nos, itidem, depascimur aurea dicta.

---

Art. I.—*An Address, delivered before the Medical Society of New-York, at their Anniversary Meeting, at the Capitol, in the City of Albany, February* 6, 1821, *on the Functions and Diseases of the Liver.* By John Stearns, M.D. President of the Society.

WHETHER we contemplate the liver as a gland or as a viscus, and consider its magnitude, the utility of its secretions, and its extensive sympathetic connexions, we must ascribe to it an importance superior to any other in the human body. In the opinion of the ancients, it possessed still higher claims to notice. It was the centre of their circulation, and the great factory in which the blood was perfected. It is, therefore, highly important that the functions of this organ and its secretions, and the diseases to which they are incident, should be correctly understood.

Since the modern improvement in physiological science, the preparation and secretion of bile are believed to constitute the chief use of this gland. Its *modus operandi* has long been a subject of inquiry; but, so minute and obscure are the operations of glandular secretions, that they will, probably, for ever elude any successful investigation. We can only trace the formation of bile to the venous blood, conveyed to the liver by a singular union of the veins of the chylopoïetic and abdominal viscera, in a vessel denominated vena portarum. As this is the only instance of the distribution of venous blood through a gland for the purpose of secretion, we necessarily impute to it the acquisition of new properties, during its passage through other glands and viscera, which renders it better qualified for the preparation of bile. The peculiar nature of these changes has hitherto eluded the researches of the chemist: neither have his experiments succeeded in deciding the long-agitated question, whether the fluids secreted by the glands are entirely new, or had previously existed in the blood.

The hepatic arterial blood has generally been considered as destined exclusively for the nourishment of the liver; but, from the arterial nature of the splenic blood, and the transmission of a branch of the cystic artery to the vena portæ, we infer that the arterial, with some modification, is an auxiliary to the venous in the formation of bile.

It is the common opinion of physiologists, that the cystic bladder, and its duct, are passive in the reception of bile, and that it is impelled into the duct by the *vis a tergo*, retaining precisely the same qualities with the bile from which it was derived.

Some late experiments, by Dr. Douglass, lead to a different conclusion, and illustrate several important phenomena not before explained. It appears, from these experiments, that the cystic duct acts as an absorbent, and selects from the bile in the hepatic duct its most active qualities. From this time the hepatic and cystic bile become entirely distinct in their qualities, destination, and use. The hepatic, deprived of its bitterness and pungency by the absorption of the cystic duct, becomes a bland and harmless fluid. It is now, with perfect safety, regurgitated into the stomach,—forms a chemical combination with the chyme, thereby constituting chyle for absorption, and precipitating the feces for evacution,—and is thus conveyed into the circulation, to aid in the formation of new materials for the blood and nourishment for the body.

The bitterness, pungency, and viscidity of the cystic bile, indicate qualities very different from the hepatic, and such as are peculiarly calculated to purge the intestines; which is, probably, the only purpose it is designed to answer. Its cathartic properties are increased during its retention in the cystic bladder, by the absorption of its dilute parts, and consequent inspissation. Some peculiar irritation produced upon the internal coat of the intestines, or mouth of the common duct, by the passing chyme, constipated feces, or any acrimonious fluid, induces a sudden discharge of cystic bile, to answer the exigencies of nature. But if, by any morbid action, the cystic bile should be regurgitated into the stomach, a nausea, vomiting, and deadly oppression, are instantly induced, which can be removed only by the total evacuation of a fluid so deleterious to that viscus. Effects still more destructive are produced when it is absorbed by the lacteals, and carried into the circulation. The great depression of the whole arterial system,—the slow, languid, and oppressed pulse,—the yellow skin,—and, indeed, the whole train of symptoms that immediately succeed this absorption, are so characteristic of that type of fever denominated malignant, that I am induced to consider cystic bile as an exciting cause of this species of fever. This was the favourite opinion of Hippocrates, and of his contemporary philosophers; and, had it been founded upon a knowledge of the circulation, and a proper distinction between the cystic and hepatic bile, they might have erected a system that would have resisted the wreck of time and the sophistry of succeeding theorists. May not fever owe its different degrees of malignity to the difference in irritation which the gradations of intensity in the cystic bile produce on the arterial system? A judicious investigation of this subject may shed light on the theory of fever. The deleterious qualities of the cystic bile are proved by the experiments of Dr. Munro, who caused the immediate death of an animal by injecting it into the stomach.

We may, therefore, safely infer, that it never exists in the stomach or blood-vessels without producing symptoms of the most dangerous tendency; while we have satisfactory evidence that hepatic bile abounds in both. This distinction may elucidate the common opinion, so incompatible with daily experience, that bile is never found in the stomach without inducing disease.

When the liver is preternaturally excited, and the bile too rapidly secreted to have its due proportion of active qualities absorbed by the cystic duct, and is thus conveyed into the circulation, it induces a febrile irritation, which can be cured only by restoring the liver to its healthy and natural secretions; but which may ultimately terminate in the ejection of bile imperfectly formed, and occasionally mixed with the dark portal blood. This is denominated black vomit, and has been frequently traced from the stomach, through the biliary ducts, to the pori biliarii in the liver.

I am aware that the ordinary occurrence of jaundice without fever, may be adduced in refutation of this theory; but, when we consider the sympathy between the liver and its ducts, it is easy to perceive that the obstruction of the latter will naturally affect the secreting process of the former; and that the pungent and active qualities of the bile may thereby be converted into a morbid and inert secretion, that will be absorbed and conveyed into the circulation with effects much less injurious to the system.

The yellow aspect of the skin in some bilious and malignant fevers, proceeds from an increased absorption of bile by the lacteals, and not from its obstruction in the ducts, and consequent reversion into the blood, as in jaundice. The difference in the colour of the skin in these two diseases, and the appearance of bile in the intestines in the former, and not in the latter, afford strong grounds for this opinion.

The distinctive properties which I have assigned to the hepatic and cystic bile, obviate the difficulties in considering it of an excrementitious or secretory character. While the former enters the circulation, and, in common with other secreted fluids, performs important uses, the latter, like other cathartics, is destined for excrementitious evacuation. This opinion is fortified by the yellow colour of the serum, without any of the bitterness of the cystic bile; and also by the nature, use, and properties, of the bile in the fetus. The latter is entirely hepatic, and destined exclusively for the nourishment of the fetus: and, even admitting its gradual absorption in the gall-bladder, there is nothing of that sympathetic intestinal irritation necessary to invite its discharge. The changes which immediately succeed the birth, from respiration and the deglutition of new food, induce that species of irritation which procures the first discharge of that cystic bile, which is necessary to commence, and for ever after to perpetuate, the peristaltic motion of the intestines. It is to the inadequacy of this irritation, or to its protracted operation, that the bile often becomes stagnant in the liver; producing, by its absorption, that icteric appearance so common in early infancy. The experiments of Dr. Douglass have contributed much towards the definitive settlement of this question. He ascertained the secretion of bile, every twenty-four hours, to amount to twenty-nine ounces, and was never able to discover more than five ounces among the evacuations during the same time: consequently, twenty-four ounces are daily absorbed and carried into the circulation.

The common opinion of physiologists, that the gall-bladder is entirely passive, and that the bile is caused to flow by the distention of the stomach after a full meal, may be included among those errors which the authority of names, and the prejudice of education, have conspired to sanction. Upon this principle, every distention of the stomach, by flatus or other causes, and every prone posture of the body, would empty the gall-bladder, as a washer-woman empties her bucket of water. Neither can I believe that the agitation excited by vomiting, or any other means, can contribute to the same effect. All prescriptions founded upon this principle alone must, therefore, be fallacious and inert. To reduce these important operations to such mere mechanical principles, is derogatory to that wisdom which formed our bodies, and animated them with living souls. And it is a subject of no ordinary surprise, that this theory should have been sanctioned by the learned physiologists of every age. A correct view of the economy of nature in the living body will conduce to the subversion of these false notions, and to the substitution of a theory more rational and consistent.

The whole animated machinery of man combines a system of causes and effects, mutually connected and mutually dependent, and deriving its principle of action from a self-moving power within. We can, therefore, never explain the operation of a single isolated part, without reference to its relations and connexions with the whole. It is with a view to this connexion that we admit the existence of a principle denominated sympathy or association, which the liver with the stomach, and intestines with the gall-bladder, possess in an eminent degree. With a full knowledge of this fact, can we divest these viscera of vitality, and ascribe their organized motions, secretions, and the discharge of bile, to a mere mechanical operation of passive inanimate matter?

When the stomach has received its supply of food, and commenced the process of digestion, the associate action of the liver is excited by their sympathetic connexion, for the purpose of supplying bile, which is always proportioned in quantity to the action of the digestive organs. The same principle promotes the secretion of the gastric juice, when nourishment is taken into the stomach, and also causes the saliva to flow at the sight of desirable food. Bichat's experiments prove that the pylorus remains closed during digestion; and that, when the chyme is discharged at this passage, the bile is at the same time poured out from the duct.

This subject might be pursued, and facts adduced in its support, from the associate operation of every organ in the human body. But the principle is too well known to need that extensive illustration of which it is susceptible, and which requires only to be applied to the stomach, liver, and their appendages, to render this theory of their respective operations perfect and consistent. The liver is powerfully excited, and the bile copiously secreted, by the continued exposure to heat. Hence, the occurrence of autumnal bilious fevers is always proportioned to the intensity and duration of the heat of the preceding season.

Two important objects, as conducive to health, are attained by this increased discharge of bile:—the food is more rapidly digested, the feces more speedily evacuated, and their putrescent tendency more effectually counteracted. Highly injurious effects would probably result from the long exposure of either to such intense heat, should their operations be subjected to the delay of colder regions. It is, therefore, necessary that the residents of tropical climates should regulate their ingesta and regimen, so as to promote the formation of new blood, sufficient to supply the loss occasioned by the increased secretion of bile. The stimulants usually resorted to in those climates increase this secretion, without augmenting the pabulum from which the blood is derived. The blood thence becomes impoverished, the bile imperfect and acrid; and fluxes, fevers, and an alarming train of morbid affections, immediately succeed.

This theory is proved by daily experience, and the diseases and premature deaths of those who migrate to tropical climates, and fall victims to their imprudence soon after their arrival.

The indeterminate use of the spleen, and the tributary aid which it yields to the liver in the formation of bile, render it necessary to bestow a few remarks upon this organ. The capacity of its blood-vessels, the red colour of its blood, and its general aspect, gave origin to the ancient opinion, that the chief use of the spleen was to impart the red colour to the blood. But, when the liver and spleen were found to be affected at the same time with similar diseases, and an unusual quantity of blood conveyed from the latter, by the splenic vein, into the vena portarum, the former yielded to the more correct opinion, that the spleen was subservient to the liver in the formation of bile.

Dr. Rush considered the spleen as merely designed to receive the superfluous blood, when its preternatural accumulation in the vessels might hazard the safety of other parts more sensible to its effects; and therefore denominated it the waste-gate of the system.

When we view the anatomical structure of the spleen, the magnitude of its blood-vessels, their tortuous and minute ramifications, and its occasional enlargement, when distended with blood, without sustaining the least injury, we are inclined to adopt the opinion of Dr. Rush: but, that this should constitute its only use, appears inconsistent with the laws of the animal economy,—the important connexion of its blood-vessels with those of the liver,—the relative situation of these organs, their mutual sympathy,—and the general opinion of physiologists. These considerations afford strong grounds to believe that the spleen becomes a reservoir for the occasional accumulation or diminution of blood in that viscus, according to the requisitions of nature, for the supply of bile.

Every excitement of the liver, by the presence of food in the stomach, by contagion, by miasmata, or by any violent exercise, fills the spleen with blood, and produces a correspondent secretion of bile.

If no reservoir of blood had been provided for such emergent calls, the secretion of bile would have been diminished at the very time when its copious discharges were most needed. Those stimuli, therefore, which increase the secretion of bile also accelerate the circulation, and fill the spleen with blood.

If this reservoir had been located in those organs which have other functions to perform, their operations must have been occasionally interrupted by an excess or defect of blood. The wisdom of the Creator is, therefore, conspicuous in providing an organ whose operations are exclusively destined to this end.

[To be continued.]

---

### Art. II.—*An Analysis of the Roots of Black Hellebore.* By MM. Feneulle and Capron.

The examination of the roots of the white hellebore, for which we are indebted to Messrs. Pelletier and Caventon, have suggested to us the propriety of laying before the public the analysis of another species of the same plant. The *helleborus hyemalis*, or winter hellebore, has already been the subject of Mr. Vauquelin's experiments; but the black hellebore, as far as we are acquainted with the fact, has never been analyzed. These two species are of the same genus, and belong to the family of the *renonculaceæ*, which has often furnished materials for analytical inquiries; while the white hellebore, on the contrary, belongs to the family of the *colchiceæ*. It was natural, therefore, for us to feel desirous of knowing, how far a similarity of names might accord with a similarity of produce in each of those plants.

Mr. Vauquelin's experiments leave not a doubt that the activity of the winter hellebore resides in the oil, or rather in a peculiar principle contained in the oil, of that plant. Thus far our own experiments agree with those of that eminent chemist; and we are convinced that no vegetable alkali, such as have lately been discovered in other plants, exists in the black hellebore; the activity of which cannot be attributed to any specific alkaline principle.

ANALYSIS.

1. A small portion of black hellebore, reduced to a coarse powder, was subjected to the action of rectified ether, several successive times; during which operation, the ether assumed a yellowish tint. The different liquids were then mixed together, filtered, and distilled to the reduction of one-half. Nothing remarkable was observed in the produce of distillation, and it was, consequently, neglected. The residue in the retort was placed in a capsule, and suffered to evaporate slowly in the open air; where, after some time, its surface became covered with a fatty substance, of a yellowish-brown colour, respecting which we shall speak hereafter.

2. The roots which had served for the preceding experiment were boiled in alcohol of the density of 36°, when the liquid assumed a brown colour. Being filtered while boiling, it deposited, on cooling, a white, fusible, and tasteless substance, which we ascertained to be wax. The alcoholic liquors were distilled, and evaporated to dryness. The produce had a soapy feel; a bitter astringent matter was obtained from it by boiling it in water, and a residue left, darker than that obtained by ether, consisting of oil and resin. The bitter astringent substance separated by boiling water from the alcoholic extract, was added to a decoction of fresh roots of hellebore; of which we shall speak presently.

3. A fresh quantity of the roots was introduced into a glass retort, with some distilled water, and suffered to macerate for some time; after which it was submitted to distillation. The produce had a nauseating smell, was transparent, exerted no action on turnsol, gave a white precipitate with the acetate of lead, and a brown one with nitrate of silver. This root, therefore, appears to contain a volatile oil.

4. The decoction, filtered boiling, deposited a small quantity of the brown matter, which re-dissolved in hot water, and was again added to the decoction from which it had separated. The filtered liquid reddened litmus paper; white precipitates were thrown down from it by muriate of barytes, oxalate of ammonia, acetate of lead, and nitrate of silver. No traces of starch could be discovered by means of iodine. Persulphate of iron struck a black precipitate; gelatine occasioned no change.

5. To the decoction, in which the brown matter separated by water from the alcoholic extract had been mixed, an excess of acetate of lead was added; the abundant precipitate thrown down on this occasion was repeatedly washed and diluted with water, and a current of sulphuretted hydrogen passed through it; the liquid was afterwards filtered, and evaporated to the consistence of extract. This, being treated by alcohol, gave an acid matter of a brown colour, striking a black precipitate with persulphate of iron; and some crystals, which were found to be gallic acid.

6. Alcohol gave a white deposit, which, although washed repeatedly in the same liquid, reddened litmus paper, and precipitated the salts of peroxide of iron black. When burnt, it left a white cinder, which effervesced with acids, and formed carbonate of lime. The portion, insoluble in spirits of wine, was oxygallate of lime.

7. Acetate of lead, in forming the precipitate of which we have spoken, (5 and 6,) almost entirely discoloured the liquid. After decomposing the excess of this salt by means of a fresh current of sulphuretted hydrogen, an extract was obtained, from which alcohol separated a mucous substance, and dissolved a brown matter, which, treated with decarbonized magnesia, produced no vegetable alkali. To prevent error, we subjected the roots of hellebore to the direct action of diluted sulphuric acid. The mixture was boiled for four hours; the liquid was saturated with caustic lime, care being taken to add an excess of the latter, and the residue washed in cold distilled water. When dry, it was re-dissolved in boiling alcohol; but no trace of vegetable alkali, such as we were looking after, could be perceived.

The brown matter, mentioned in the preceding experiments, possesses the properties common to vegetable bitters, without appearing to act as the principle on which depend the poisoning qualities of the hellebore.

OF THE FAT MATTER.

8. The colour of this substance is of a brownish yellow; its taste is acrid, and only manifests itself, in all its intensity, at the expiration of several minutes. Its consistence is soft; ether dissolves it readily at the common temperature; and a smaller quantity of it is dissolved by alcohol. It reddens tincture of litmus strongly. The experiments of Messrs. Pelletier and Caventou guided us in our essays. When the oil of black hellebore is boiled for an hour in water, it appears to lose part of its taste and acidity. These properties are still farther impaired, when caustic magnesia, or potash, are added to the oil of hellebore; and are completely destroyed by a long exposure of the latter to the air.

9. A portion of the oil (*matière grasse*) of the black hellebore was saponified by means of caustic potash; the produce dissolved in water, and decomposed by tartaric acid. The filtered liquid was distilled; when strong indications of acid were observed. Owing to the very small quantity of the produce, we did not attempt to insulate the acid in question; but we satisfied ourselves as to its saturating ammonia, potash, soda, and magnesia, forming with each soluble salts. This acid, possessing a peculiar smell, appears to us to be analogous to that which Messrs. Pelletier and Caventou discovered in the grains of the *jatropha multifida*, and to which those chemists attribute the active properties of that plant.

10. A small quantity of the roots of black hellebore, previously bruised, was macerated in distilled water for several hours. The infusion was then boiled, but no albuminous matter could be discovered. The liquid was next evaporated to dryness; potash disengaged much ammonia. Another portion of the extract, treated with sulphuric acid, did not manifest the presence of any acetate.

11. The roots, treated with ether, alcohol, and water, (1, 2, 3,) were boiled in a solution of sub-carbonate of potash for some minutes, and the liquid was saturated with muriatic acid, when a considerable quantity of *alumine* was deposited.

12. The residue obtained by burning some of the roots of hellebore in a platina crucible was found to consist of subcarbonate, sulphate, and muriate, of potash; of subcarbonate and phosphate of lime; oxide of iron; and silica.

From the preceding experiments, we deem ourselves authorized to conclude, that the active properties of the black hellebore is due to a peculiar acid combined with the oil of that plant, to which it also imparts its peculiar smell.

The results of the chemical analysis of the black hellebore are, therefore, as follow:

1. A volatile oil.
2. A fixed oil.
3. A Resinous matter.
4. Wax.
5. A volatile acid.
6. A bitter principle.
7. A mucous principle.
8. Alumine.
9. Gallate of potash, and oxygallate of lime.
10. An ammoniacal salt.

*Journal de Pharmacie, Nov.* 1821.

☞ To those who may be inclined to consult the original, it may not be improper to mention, that several errors of the press occur in it; the more important of which are "*gelate* acide de chaux," for "gallate acide de chaux;"—"*le* saponifiait," for "se saponifiait;"—"*ulmine*," for "alumine."—A. B. G.

ART. III.—*Observations on the Connexion of Electric and Magnetic Phenomena.*

No discovery has, for a long time, so strongly excited the attention of the philosophic world, as that of the magnetic phenomena belonging to the voltaic apparatus: we shall, therefore, endeavour to give our readers a short statement of what has been done in this department of scientific inquiry.

1. If the extremes of a voltaic battery, (we will suppose it to consist of twenty pairs of eight-inch plates,) be connected by a platinum wire, it becomes heated, and, if of sufficiently small diameter, it suffers ignition. Let us suppose such a wire, W, lying upon the supports P and N, which represent the positive and negative conductors of the active voltaic apparatus, P being connected with the first zinc plate, and N with the last copper plate; upon bringing the north pole of a common magnetic needle *below* and at a right angle to the platinum wire, it will be repelled or driven downwards: if we now remove the needle, keeping it in the same position, so that its north pole may be *above* the platinum wire, it will then be attracted towards it. If the electric poles be reversed, these phenomena will also be reversed.

If we suppose the conjunctive platinum wire to be vertical, instead of horizontal, and in that position approach it with either end of the magnetic needle, the needle will *oscillate*, but will not be permanently attracted or repelled by any part of the conjunctive wire.—Professor OERSTED.

2. If a small steel bar be attached to the conjunctive wire, and parallel to it, it does not become a polar magnet; but, if it be attached transversely, it does become polar; and it becomes north and south, or south and north, according to the direction of the supposed electric current traversing the conjunctive wire, according as one or the other end of it is positive or negative. Thus, supposing W to represent the platinum conjunctive wire of the voltaic apparatus, and N S a wire of iron attached transversely to it, the latter becomes permanently magnetic.—Sir H. DAVY.

3. If we suppose a second conjunctive wire parallel to, and similarly situated with, the first, (as in this figure,) those wires will *attract* each other; but, if one conjunctive wire be traversed by the electric fluid in one direction, and another in an opposite direction, (as in the following wood-cut,) those wires will *repel* each other. In this circumstance, the dissimilarity of the electro-magnetic and of simple electric phenomena is observed; for, bodies similarly electrified repel each other, and, dissimilarly electrified, attract each other; but here the horizontal wires, similarly electro-magnetized, attract; and, dissimilarly electro-magnetized, repel each other.—M. AMPERE.

4. The shock of a Leyden jar, or battery, passed through a wire, confers upon it, at the moment of its passage, properties precisely similar to those of the voltaic apparatus.

To render a steel bar magnetic, it is not necessary that it should *touch* the conjunctive wire, to which it is attached at right angles; for the electro-magnetic influence is conveyed to some distance, and is not excluded by the interposition of a plate of glass, of metal, or of water. —Sir H. DAVY.

5. The phenomena exhibited by the electro-magnetic, or conjunctive wire, may be explained upon the supposition of an electro-magnetic current passing round the axis of the conjunctive wire; its direction depending upon that of the electric current, or upon the poles of the battery with which it is connected.—Dr. WOLLASTON.

In the above figure, such a current is represented in two sections at right angles to the axis of the wires, when similarly electrified; from which it will be apparent that the north and south powers, meeting, will attract each other.

In the following figure, the sections of the wire are shown dissimilarly electrified, by which similar magnetic powers meet, and consequently occasion a repulsion.

*Journal of Science*, vol. x.

## Figures and Tables

**Figure f1:**
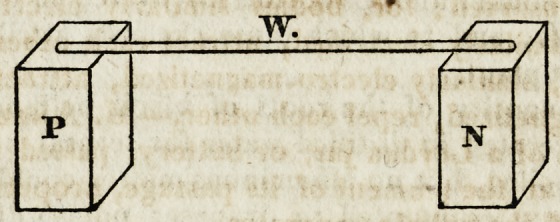


**Figure f2:**
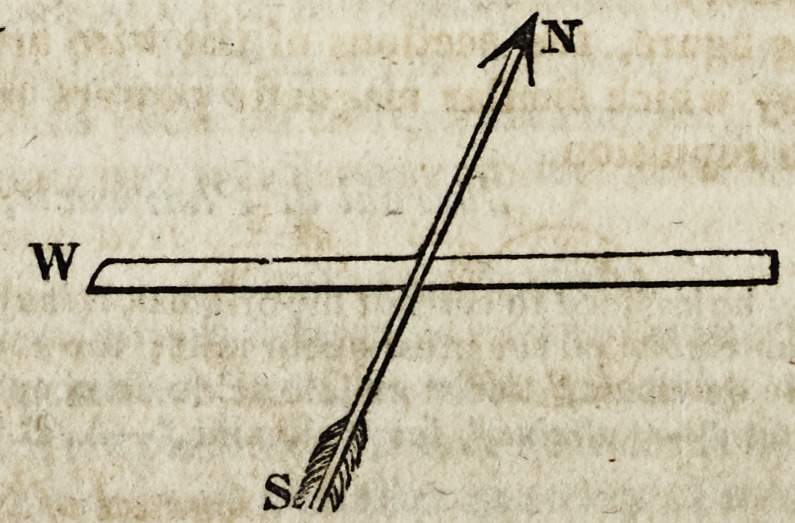


**Figure f3:**
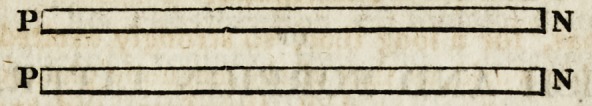


**Figure f4:**
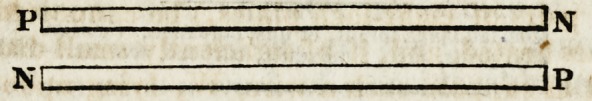


**Figure f5:**
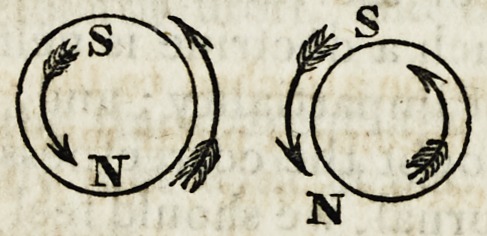


**Figure f6:**